# Knowledge and malaria treatment practices using artemisinin combination therapy (ACT) in Malawi: survey of health professionals

**DOI:** 10.1186/1475-2875-10-279

**Published:** 2011-09-22

**Authors:** Linda V Kalilani-Phiri, Douglas Lungu, Renia Coghlan

**Affiliations:** 1 College of Medicine, University of Malawi, Private Bag 360, Blantyre 3, Malawi; 2 Daeyang Luke Hospital, P.O. Box 30330, Lilongwe 3, Malawi; 3 Medicines for Malaria Venture, Geneva, Switzerland

## Abstract

**Background:**

Malaria still remains a life-threatening disease worldwide causing between 190 and 311 million cases of malaria in 2008. Due to increased resistance to sulphadoxine-pyrimethamine (SP), the Ministry of Health in Malawi, as in many sub-Saharan African countries, changed the malaria treatment policy to use artemisinin-based combination therapy (ACT). In order to optimize the correct use of this drug, and protect against the development of the parasite's resistance, it is important to assess the knowledge and practices of medical practitioners on the use of ACT and its impact on adherence to new treatment policy guidelines.

**Methods:**

A cross-sectional survey was conducted to assess the knowledge and perceptions of Malawian medical doctors and pharmacists on the use of ACT and the drivers of treatment choice and clinical treatment decisions. Medical doctors and pharmacists who are involved in managing malaria patients in Malawi were recruited and a self-administered questionnaire was used to obtain information on socio-demographic characteristics of the study participants, knowledge on ACT, source of information on ACT and methods used to decide on the treatment of patients with malaria.

**Results:**

Most of the participants (95.7%) know at least one form of ACT, 67.4% reported that different forms of ACT have different characteristics, 77.3% reported that there are special formulations for children. The most commonly mentioned ACT was artemether-lumefantrine (AL), by 94.6% of the participants and 75.0% of the participants indicated that they prefer to prescribe AL. 73.9% of participants had ever received information on ACT. However, only 31.5% had received training on management of malaria using ACT. There were 71.7% respondents who had heard of ACT causing side effects. Only 25.0% of the participants had received training on how to report SAEs.

**Conclusion:**

It was found that most of the participants know about ACT and treatment guidelines for malaria. However, most of the participants have not received any training on how to use ACT and how to report adverse effects arising from the use of ACT. There is need for more training of health care professionals to ensure correct and effective use of ACT.

## Background

Malaria still remains a major public health problem worldwide. It is estimated that between 190 and 311 million cases of malaria occurred in 2008 (1). Malaria and its complications are controlled by preventing infection, prompt diagnosis and effective treatment [1,2]. Due to the widespread resistance to chloroquine and sulphadoxine-pyrimethamine (SP), the World Health Organization (WHO) currently recommends the use of artemisinin-based combination therapy (ACT) for the treatment of *Plasmodium falciparum *infection, and *Plasmodium vivax *infection resistant to chloroquine. Artemisinin derivatives rapidly decrease parasite density and gametocyte levels in treated patients, and, therefore, greatly reduce the level of infectiousness, and transmission of resistant strains [3]. There are several forms of ACT that are currently being used for treatment of malaria including artemether-lumefantrine (AL), artesunate-mefloquine, artesunate-amodiaquine, artesunate-sulphadoxine-pyrimethamine (SP), and dihydroartemisinin-piperaquine (DHA-PQ)[4].

Currently, most countries in sub-Saharan Africa have switched to using ACT as the first- and/or second-line of treatment of malaria [5]. The uptake of the new treatment policies has faced several challenges including inadequate diagnostic facilities and inappropriate use of drugs [6]. To ensure that new treatment guidelines and recommendations are effectively utilized, a systematic involvement of all the key stakeholders (including policy-makers, media, practitioners and the general public) at all stages of implementation is crucial [7]. Only a few studies have examined the impact of knowledge and practices of medical practitioners on the use of ACT and adherence to new treatment policy guidelines in countries that have adopted the use of ACT. One study found that despite high level of awareness of a new malaria treatment policy, a significant percentage of clinicians (76.2%) reported continued use of SP [8]. Another study found that there was lack of involvement of the pharmaceutical personnel working in the private pharmacies, from the preparation of new malaria treatment guidelines to their implementation, and this contributed to their poor knowledge and skill on how to correctly dispense the medicines [9].

Three years have now passed since Malawi changed the treatment policy to ACT. The first-line treatment for uncomplicated malaria is AL and the second-line is amodiaquine-artesunate. Recommended treatment for severe malaria is quinine. For pregnant women, AL is used for treatment of uncomplicated malaria in the second and third trimester whilst quinine is used for treatment of uncomplicated malaria in the first trimester of pregnancy and also for severe malaria in all trimesters. Accordingly, there is need to assess the knowledge and practices of doctors and pharmacists as they are key health care personnel influencing the dispensing of anti-malarials. This has an impact on the risk for development drug resistance if proper guidelines are not followed. This information would be useful for guiding discussions to expand access to, affordability and appropriate use of ACT, notably in a period of increasing therapeutic options, and a shift to confirmed case management of malaria.

## Methods

This was a cross-sectional survey that was conducted in Malawi between April and September 2010. Using a list obtained from the Malawi Medical Council and the Pharmacy Medicines and Poisons Board (PMPB), a random sample of medical doctors and pharmacists who manage patients with malaria or dispense antimalarials in the public and private sector were recruited. Doctors and pharmacists who were not involved in managing malaria patients or dispensing anti-malarials and participants who did not provide consent were excluded from the study. The required sample size was 112 with 80% power, assuming that 185 doctors and pharmacists are registered to practice in Malawi, 80% of these manage patients with malaria and that 70% had ever heard of ACT. At the time the study was conducted, there were 205 registered doctor and pharmacists, and responses were obtained from 92 participants giving a 99% power (posthoc) for the study. The study participants were notified about the objectives of the study via email, a phone call or in person at any opportune time and were asked for verbal consent to participate in the study. After providing consent, the participants were given the questionnaire in person or via email or post (Additional file 1). Data collected included socio-demographic characteristics of the study participants, knowledge on ACT, source of information on ACT and methods used to decide on the treatment of patients with malaria. The participants either emailed back the questionnaire or it was collected from their work place or at relevant meetings. To ensure confidentiality, each questionnaire was assigned a study number and the participants were not required to fill in their name to anonymize the data. Where identifying information was indicated on the questionnaire, it was removed during data entry and a link file was used to identify the participants. Participants who did not return the questionnaire were contacted by phone or email at least three times and if there was still no response, an attempt was made to visit them at their work place to obtain the questionnaire. This study was approved by the College of Medicine Research and Ethics Committee; protocol number P.05/10/941.

The data was double-entered into Microsoft Excel, cleaned and analysed using STATA version 10. Descriptive analysis of categorical data was done using frequencies and percentages while continuous data were described using means, standard deviations, medians and interquartile ranges. The percentages were calculated based on the total number of responders per question. Comparison of categorical data was performed using the Pearson Chi-square test or Fisher's Exact test if necessary. Responses to open ended questions were grouped into common themes.

## Results

### Demographic characteristics of study participants

Data was obtained from 44.9% (92/205) of the doctors and pharmacists listed on the Malawi Medical Council and PMPB register. Most of the respondents were male (62.0%, n = 57/92), general practitioners (72.2%, n = 65/90) with a median age of 32 years (Interquartile range (IQR) 26-40 years). The median duration the participants had been working in their profession was 3 years (IQR 1-9 years). Further information on the characteristics of the respondents is shown in Table 1.

**Table 1:** Demographic characteristics of the study participants

Characteristic	N = 92
Age, median (IQR), years	32 (26-40)
Male (%)	57 (62.0)
Primary profession (%)	
General practitioner	65 (72.2)
Medical specialist	17: (18.9)
Pharmacist	5 (5.6)
Pharmacy Technician	2 (2.2)
Pharmacy Assistant	1 (1.1)
Work sector (%)	
Private	28 (30.4)
Public	59 (64.1)
Non-governmental organization	5 (5.4)
Location of practice (%)	
Urban	76 (82.6)
Rural	16 (17.4)
Duration of practicing, median (IQR), years	3 (1-9)
Number of patients seen per week, median (IQR)	140 (70-210)
Number of malaria patients seen per week, median (IQR)	35 (10-70)

### Malaria diagnosis practices and knowledge on malaria treatment guidelines

Most respondents reported that they diagnosed malaria using microscopy (73.1%, n = 60/82), some used both Rapid Diagnostic Tests (RDTs) and microscopy (25.6%, n = 21/82). Only 1.2% (n = 1/82) reported relying on RDTs alone for diagnosis. Almost all of the respondents (84.1%, n = 69/82) also used clinical symptoms when diagnosing malaria. Factors contributing to the choice of test used for malaria diagnosis included availability of tests (49.0%, n = 24/60), patient symptoms (26.5%, n = 13/60) and cost of the test (10.2%, n = 5/60).

Most the participants knew the first line treatment for uncomplicated malaria [children (94.6%, n = 87/92) and adults (96.7% (n = 89/92)] and for severe malaria [children (96.7% (n = 89/92) and adults (97.8% (n = 90/92)]. However, not many participants knew the treatment guidelines for pregnant women with only 40.0% (n = 34/85) indicating the correct treatment guidelines for uncomplicated malaria and 82.6% (n = 76/92) for severe malaria. Most of the participants [79.7% (59/74)] reported that they used treatment guidelines when managing patients with malaria.

### Malaria treatment practices

Most of the participants (70.7%, n = 65/92) reported that the type of ACT that they prescribe varies depending mainly on the age of the patient (72.3%) and the clinical picture of the patient (64.6%). Most of the participants also reported that they preferred to prescribe AL (75%, n = 69/92) followed by artesunate-SP (8.7%, n = 8/92), artesunate-amodiaquine (5.4%, n = 5/92), and DHA-PQ (1.1%, n = 1/92). Reasons for prescription preferences included following government treatment policy (79.7%, n = 55/69), therapeutic efficacy (55.1%, n = 38/69) and cost of the drugs (36.2%, n = 25/69).

### Knowledge on artemisinin combination therapy

Almost all of the participants (97.8%, n = 90/92) had heard about ACT and most were able to mention at least one type of ACT: one type of ACT (40.2%, n = 37/92), two types of ACT (29.4%, n = 27/92), three types of ACT (13.0%, n = 12/92), four types of ACT (10.9%, n = 10/92) and five types of ACT (2.2%, n = 2/92). However, some of the participants (40.2%, n = 37/92) did not differen-tiate between a trade and a generic name and, therefore, repeated the names of ACT they know. Pharmacists/Pharmacy Technicians/Pharmacy Assistants were more likely to mention three or more types of ACT compared with doctors/medical specialists, p < 0.001. Most of the participants (92.4%, n = 85/92) indicated that AL is available in Malawi and almost all participants (93.5%, n = 86/92) have prescribed AL (Table 2).

**Table 2:** Knowledge of types of ACTs and availability in Malawi

	Known	Available	Dispensed ACTs
	**ACTs, n (%)**	**ACTs, n (%)**	**ACTs, n (%)**

Artemether- Lumefantrine	87 (94.6)	85 (92.4)	86 (93.5)
Artesunate Amodiaquine	35 (38.0)	31 (33.7)	10 (10.9)
Artemisinin-SP	18 (19.6)	18 (19.6)	14 (15.2)
Dihydroartemisinin-piperaquine	13 (14.1)	6 (6.5)	5 (5.4)
Artesunate-Mefloquine	9 (9.8)	4 (4.3)	2 (2.2)
Pyronaridine-Artesunate†	6 (6.5)	0	0
Dihydroartemether/Piperidyl Quinine	4 (4.3)	3 (3.3)	2 (2.2)
Piperaquine-Artesunate	2 (2.2)	0	0
Chloroproguanil-Dapsone Artesunate	2 (2.2)	0	0

†This product is still in development and is therefore correctly indicated as not being available and not yet being prescribed

Most of the participants (67.4%, n = 62/92) indicated that different types of ACT have differences in cost (75.8%, n = 47/62), appearance (64.5%, n = 40/62) and mode of action (43.6%, n = 27/62), (Table 3). There was no significant difference in the responses between medical doctors and pharmacists, p > 0.05.

**Table 3:** Knowledge on difference in ACTs and types of formulations

Characteristic	N (%)
Type of formulation or packaging*	
Tablet	23 (37.7)
Syrup	16 (26.2)
Dispersable	9 (14.8)
Powder	6 (9.8)
Suspension	4 (6.6)
Characteristics of ACTs**	
Price	47 (75.8)
Appearance	40 (64.5)
Therapeutic efficacy	38 (61.3)
Mode of action	27 (43.6)
Quality	26 (41.9)
Individuals who can use them	25 (40.3)
Safety	18 (29.0)
Ease of use	3 (4.8)
Dosage	3 (4.8)
Manufacturer	2 (3.2)
Side effect profile	1(1.6)

*This question was for 62 respondents who indicated that there are differences in paediatric formulations of ACTs

**There were 62 doctors who indicated that there are differences in the characteristics of ACTs

Most participants (70.7%, n = 65/92) indicated that there are types of ACT spe-cifically manufactured for children. They noted that a paediatric formulation is available for AL (89.2%, n = 58/65), artesunate-amodiaquine (10.8%, n = 7/65), artesunate-SP (7.7%, n = 5/65), dihydroartemether/piperidyl-quinine (1.5%, n = 1/65) and pyronaridine-artesunate (1.5%, n = 1/65). The participants also indicated that there are different types of packaging of the children's medication including blister packs 6.6% (n = 4/61) and sachets 1.6% (n = 1/61) and formulations including tablets, powder and suspension (Table 3).

### Side effects of ACT and pharmacovigilance

Most of the participants (71.7%, n = 66/92) reported that they had heard of ACT causing side effects. Most of the side effects reported were for AL and these included nausea (32.8%), headache (25.0%) and vomiting (21.9%) (Table 4). The only other type of ACT that was reported to cause side effects was DHA-PQ (skin rashes) and artesunate-SP (nausea, vomiting, diarrhoea, Steven-Johnson syndrome and anorexia). Most of the participants (77.2%, n = 71/92) indicated that they never had a severe adverse event (SAE) reported as a result of patients taking ACT. However, a few participants had received SAE reports ranging from 1-10 over a period of 1 to 12 months. Nearly 40% of the participants (38.0%, n = 35/92) knew where to report SAEs, while only 25.0% (n = 23/92) had received training on how to report SAEs.

**Table 4:** Reported side of effects of taking Artemether- Lumefantrine

Side effect	N* (%)
Nausea	21 (32.8)
Headache	16 (25.0)
Vomiting	14 (21.9)
Abdominal pain	11(17.2)
Skin Rash/urticaria/pruritus	9 (14.1)
Lethargy/Myalgia/Arthralgia	9 (14.1)
Dizziness	7 (10.9)
Diarrhoea	8 (12.5)
Cardiac arrhythmias	5 (7.8)
Anorexia	4 (6.3)
Sleep disorders	4 (6.3)
Elevated liver enzymes/hepatotoxicity	3 (4.7)
Fetotoxicity	3 (4.7)
Palpitations	4 (6.3)
Weakness/fatigue	2 (3.1)
Indigestion	2 (3.1)
Anaemia	1 (1.6)
Jaundice	1 (1.6)
Oral sores	1 (1.6)
Hallucinations	1 (1.6)
Renal toxicity	1 (1.6)

* This only includes 64 respondents who had heard of side effects of ACTs

### Source of information on ACT

Most of the participants (73.9%, n = 68/92) reported that they have ever received information on ACT. Participants reported multiple sources of information including books (50.0%, n = 34/68), peer-reviewed journals (45.6%, n = 31/68), drug formulary (51.5%, n = 35/68), government documents (64.7%, n = 44/68), from the internet (17.6%, n = 12/68), brochures (2.9%, n = 2/68), scientific meetings (4.4%, n = 3/68) and from colleagues 1.5% (n = 1/68). Only 32 (34.8%) of the participants had received information on ACT from pharmaceutical companies, including Novartis (40.6%, n = 13/32), Dafra (6.3%, n = 2/32), GlaxoSmithKline (GSK) (6.3%, n = 2/32) and Intermed Pharmaceutical (6.3%, n = 2/32), Medinomics (3.1%, n = 1/32). There was one participant (3.1%) who obtained information from Medicines for Malaria Venture (MMV), a public-private-partnership organization that develops new anti-malarial medicines in collaboration with pharmaceutical companies for public health aims. Furthermore, only 29 (31.5%) of the participants have received training on management of malaria using ACT mostly from the Ministry of Health (MoH) (55.2%, n = 16/29) or the National Malaria Control Programme (10.3%, n = 3/29).

## Discussion

This study was conducted to assess the knowledge of health care professionals on ACT and their treatment practices using ACT. Most of the participants (95.7%) knew at least one type of ACT, 67.4% reported that different forms of ACT have different characteristics, 77.3% indicated that there are special formulations for children and 71.1% had heard of side effects caused by ACT. Although most of the participants had heard about ACT, 40.2% were not able to differentiate between trade and generic names of the drugs. As such, they listed a drug several times using different trade names assuming that they were different drugs. Furthermore, only 13.1% of the participants were able to list at least four types of ACT. Further training or providing information materials might be useful to help clarify the difference between brand names and generic or molecular names i.e. International Nonproprietary Names (INN) of ACT.

AL was the most commonly mentioned ACT by 94.6% of the participants. Other commonly mentioned ACT included artesunate-amodiaquine, artemisinin-SP and DHA-PQ. AL and artesunate-amodiaquine are the first- and second-line treatment in Malawi, respectively. Pharmacists were significantly more likely to mention at least three types of ACT compared with doctors. This could be because pharmacists manage drugs on a more regular basis than doctors. Most of the participants (67.4%) indicated that different types of ACT are different in terms of appearance, mode of action and therapeutic efficacy and 77.3% indicated that there are types of ACT that are specially formulated for children, which are packaged as tablets, syrup, powder, blister packs or suspension. Studies have shown that paediatric formulations are easier to administer to children and are better tolerated than adult formulations [10]. It is, therefore, encouraging to note that most of the participants know these formulations to ensure effective management children with malaria. However, it should be noted that artemisinin-based treatments in syrup or powder form may be less stable and are, therefore, not recommended by WHO as appropriate formulations for this class.

Only a few (31.5%) participants received training on how to manage patients using ACT. Studies in other countries have also found that few health care professionals receive training after changes in treatment policy and this has an impact on prescription practices [11,12]. Most of the participants obtained their information on ACT from books, peer reviewed journals and the drug formulary. Despite the low coverage in training, most of the participants (75.0%) indicated that they prefer to prescribe AL following government policy recommendations. It appears therefore that most of the health care professionals are following government treatment guidelines. Other reasons for prescription preferences included therapeutic efficacy, availability of drugs, possibility of use in children, price, appearance, confidence in the quality of manufacturer and treatment regimen; some of these factors have also been cited in other previous studies [13,14].

The ACT that was most commonly reported to cause side effects was AL. This could be attributed to the fact that AL is used more frequently in Malawi than any of the other types of ACT. The other types of ACT mentioned to cause side effects were DHA-PQ and artesunate-SP. It is important to note that some of the side effects that were reported such as headache, nausea, vomiting are also symptoms of malaria, and therefore might not be as a result of taking the drugs. Surprisingly, there was no mention of side effects caused by amodiaquine-artesunate, the second-line treatment for malaria in Malawi. Most of the health care professionals reported that they do not know how to report SAEs and therefore the government should consider providing more training on SAEs reporting and management as this has an impact on drug adherence.

Most of the participants knew the treatment guidelines for uncomplicated and severe malaria in children, non-pregnant adults and treatment of complicated malaria in pregnant women. However, only 40.0% of the participants knew the correct treatment guidelines for uncomplicated malaria in pregnant women. Pregnant women are one of the groups at most risk for malaria and its complications [15]. Additionally, since Malawi is a high malaria transmission area, most of the pregnant women present with asymptomatic malaria or a mild illness [16]. Therefore, with such low levels of knowledge on treatment guidelines, most pregnant women with uncomplicated would receive the wrong treatment.

Most of the doctors (73.1%) were still using microscopy to diagnose malaria. There were a few doctors who were using either RDTs only (1.2%) or RDTs and microscopy (25.6%) to diagnose malaria. Almost all of the doctors (84.1%) were also using clinical symptoms to diagnose malaria. The decision on which test to use depended on the availability of the test, costs and patient symptoms. RDTs have just been introduced in Malawi and, therefore, might not be readily available for use in all health facilities, clinics or other facilities. In most malaria endemic areas, diagnosis is done presumptively based on symptoms due to lack of equipment and microscopy expertise [17]. It is good to note that on the contrary, most of the doctors in this study diagnose malaria using a diagnostic test (microscopy or RDTs) in addition to symptoms.

The study had the following limitations. Not all participants who were approached gave responses and they might have had different characteristics from the individuals who participated in this study. Second, the questionnaires were self-administered and some participants did not fill in all the required information. Third, the study recruited medical doctors and pharmacists only and therefore the results cannot be generalized to other cadres like clinical officers, nurses and community health workers who are also involved in managing patients with malaria. There is need to conduct further studies to assess if the level of knowledge and treatment practices would be different in the other cadres not included in this study. However, despite these limitations, important information was obtained on the knowledge and practices of health professionals in Malawi on ACT.

## Conclusion

In conclusion, most of the health care professionals have heard about ACT and they know the treatment guidelines for malaria. However, only a few health care professionals have received training on how to use ACT and how to report SAEs resulting from ACT. It is important for the MOH to continue engaging health care professional to ensure correct use of ACT for proper management of malaria patients and to reduce the risk of development of resistance to ACT.

## List of abbreviations

ACT: Artemisinin Combination Therapy; SP: Sulphadoxine-Pyrimethamine; AL: Artemether-Lumefantrine; DHA: PQ-Dihydroartemisinin-piperaquine; GSK: GlaxoSmithKline; INN: International Nonproprietary Names; IQR: Interquartile Range; MMV: Medicines for Malaria Venture; MoH: Ministry of Health; PMPB: Pharmacy Medicines and Poisons Board; RDTs: Rapid Diagnostic Tests; WHO: World Health Organization

## Competing interests

The authors declare that they have no competing interests.

## Authors' contributions

LK, DL and RC designed the study; LK and DL collected the data; LK analysed the data; LK, DL and RC interpreted the data and prepared the manuscript. All authors read and approved the final manuscript.

## Supplementary Material

Additional file 1 **Study Questionnaire**. This is the self-administered questionnaire that was given to study participants. Click here for file
