# Supplementary material for: Knowledge and malaria treatment practices using artemisinin combination therapy (ACT) in Malawi: survey of health professionals
Source: Malar J. 2011 Sep 22;10:279. doi: 10.1186/1475-2875-10-279 (PMC3196928; doi:10.1186/1475-2875-10-279)
Supplement: Additional file 1 — Study Questionnaire. This is the self-administered questionnaire that was given to study participants. [file 1475-2875-10-279-S1.DOC]

# Appendix I: Study questionnaire

# Knowledge and Malaria Treatment Practices using Artemisin Combination Therapy (ACT) in Malawi- “Survey of Health Professionals in Malawi”

| **Module I: Basic Information** | | |
| --- | --- | --- |
| **No** | **Questions and Filters** | **Responses and Codes** |
| 101 | ***Gender of respondent*** | 1. Male   2. Female  |
| 102 | ***Which of the following categories describes your primary profession?*** | 1. General Medical Doctor   2. Pharmacist   3. Medical Specialist (specify)  |
| 103 | ***What are your qualifications?*** | ……………………………………………………………………..  …………………………………………………………………….. |
| 104 | ***In which sector do you work in primarily?***  **[Interviewer: If you work in more than one sector, tick all the categories corresponding to the sector you work and rank them in order of importance based on the time you contribute to the sector.** | Rank  1. Private sector…………………………...…………….  2. Public sector (government)……....…………….   3. Non-governmental organization...….………… |
| 105 | How long have you been working in this field? | ____ Years (to the nearest year) |
| 106 | How old are you? | ___|___ Years (age at last birthday) |
| 107 | In which area do you **currently** work?  **[Interviewer: If you work in both rural and urban areas, tick the category corresponding to the area where you contribute the most time.]** | 1. District / Rural area   2. Urban area  |
| 108 | How many patients in total do you see per day or week?  [**Indicate either number of patients per day or per week depending on what you think is the most accurate number**] | No. per day_...................................................  No per week………………………………….. |
| 109 | How many patients with malaria do you see per day/week?  [**Indicate either number of patients per day or per week depending on what you think is the most accurate number**] | No. per day_...................................................  No per week………………………………….. |
| 110 | How do you diagnose malaria?  [**Tick all that apply**] | 1. Rapid Diagnostic Tests  2. Microscopy  3. Clinical symptoms  4. Other  |
| 111 | If you use more than one method to diagnose malaria, how do you decide which method to use for diagnosing malaria? | ………………………………………………………………………  ………………………………………………………………………  ………………………………………………………………………  ……………………………………………………………………… |

| **Module II: Knowledge about Artemisin-based Combination Therapies (ACTs)** | | |
| --- | --- | --- |
| 201 | What is the first line treatment for uncomplicated malaria for children in Malawi? | ……….................................................................................................. |
| 202 | What is the first line treatment for uncomplicated malaria for adults in Malawi ? | ……….................................................................................................. |
| 203 | What is the first line treatment for uncomplicated malaria for pregnant women in Malawi? | ……….................................................................................................. |
| 204 | What is the treatment for severe malaria for children in Malawi? | ………..................................................................................................  ………................................................................................................... |
| 205 | What is the treatment for severe malaria for adults in Malawi? | ………..................................................................................................  ………................................................................................................... |
| 206 | What is the treatment for severe malaria for pregnant women in Malawi?   1. In the first trimester of pregnancy 2. In the second trimester of pregnancy 3. In the third trimester of pregnancy | a. ………..............................................................................................  b. ………..............................................................................................  c. ……….............................................................................................. |
| 207 | Is there a treatment policy for malaria in Malawi? | 1. Yes ……………………………….... **🡪Go to Q208**  2. No ……………………………………..**🡪Go to Q209**  **3. Don’t Know** ……………………... **🡪Go to Q209** |
| 208 | When was the current malaria treatment policy introduced in Malawi? | **__|__ : __|__: __|___|___|__**  **Day Month Year**  **Don’t Know** ………………………………………... |
| 209 | Have you ever heard of Artemisinin-based combination therapies (ACTs)? | 1. Yes ……………………………….... **🡪Go to Q210** 2. No…………………….………….....**🡪Go to Q401** |

|  | 210 | | Please provide a complete list of names of ACTs that you have heard of. | 1. ……………………………………………………………  2. …………………………………………………………….  3………………………………………………………………  4………………………………………………………………  5………………………………………………………………  6. …………………………………………………………….  7………………………………………………………………  8. …………………………………………………………….  9. …………………………………………………………….  10. ………………………………………………………….. | |
| --- | --- | --- | --- | --- | --- |
|  | 211 | | Please list the ACTs you believe are available in Malawi | 1. ……………………………………………………………  2. …………………………………………………………….  3………………………………………………………………  4………………………………………………………………  5………………………………………………………………  6. …………………………………………………………….  7………………………………………………………………  8. …………………………………………………………….  9. …………………………………………………………….  10. ………………………………………………………….. | |
|  | 212 | | Please list the ACTs you have used/dispensed during your practice. | 1. ………………………………………………….………..  2. ……………………………………………………………  3……………………………………………………………..  4……………………………………………………………..  5……………………………………………………………..  6. ……………………………………………………………  7……………………………………………………………..  8. ……………………………………………………………  9. ……………………………………………………………  10. ………………………………………………………….. | |
|  | 213 | | Do you consider that the ACTs which you listed in Q210 and Q212 have different characteristics? (e.g. in terms of safety, therapeutic efficacy, ease of use, quality etc) | 1. Yes ………………………………....**🡪Go to Q214** 2. No……………………….……….....**🡪Go to Q215** | |
|  | 214 | | How are the ACTs different?  **[Tick all the answers that apply]** | 1. Appearance……………………………………….... 2. Mode of action…………………….…………........ 3. Individuals who can use them…………........ 4. Price…………........................................................... 5. Quality…………....................................................... 6. Safety …………........................................................ 7. Therapeutic Efficacy………….............................. 8. Other (specify) …………..................................... | |
|  | 215 | | Which ACTs do you prefer to prescribe, from the lists you have provided in Q210, 211, 212 (Please provide the brand name, active ingredients / INN and the manufacturer).  **[You may indicate more than one product].** | 1. …………………………………………..………………. 2. ………………………………………………………….. 3. …………………………………………..………………. 4. …………………………………………..………………. 5. …………………………………………..………………. | |
|  | 216 | | Why do you prefer these products?  **[You may select more than one choice].** | 1. Therapeutic Efficacy……………………….... 2. Price………...…………………………………….... 3. Appearance…………………………………….... 4. Mode of action / Formulation.……..…..... 5. Individuals who can use them…………..... 6. Regimen……………………………..…………..... 7. Adapted for Children.…………..…………..... 8. Government Policy….………………………..... 9. Ease of supply……..….…………..……………..... 10. Confidence in quality of manufacturer.. 11. Other (specify) …………................................. | |
|  | 217 | | Are any of the ACTs you have listed in Q210-Q212 specially designed for treatment of malaria children? | 1. Yes ………………………………....**🡪Go to Q218**   2. No………………….…………….....**🡪Go to Q221** | |
|  | 218 | | Please list the ACTs that are specific for treatment of malaria children. | 1……………………….………………………….  2………………………………………………….  3………………….……………………………….  4…………..………………………………………. | |
|  | 219 | | Are any of the ACTs you have listed in Q215 specially formulated for consumption by children? | 1. Yes ………………………………....**🡪Go to Q220** 2. No………………….…………….....**🡪Go to Q221** | |
|  | 220 | | Please list the ACTs that are specifically formulated for consumption by children for children. | 1. ………………………………………. 2. ……………………………………….   3. ….……………………………………….  4. …………………………………………. | |
|  | 221 | | Are there any of the drugs that you have listed in Q215 specially packaged for consumption by children? | 1. Yes ………………………………....**🡪Go to Q222** 2. No………………………….…….....**🡪Go to Q2223** | |
|  | 222 | | In what form have they been packaged for use by children? | 1. ……………………………………………………………… 2. ………………………………………………………………… | |
|  | 223 | | Have you ever heard of any side effects caused by ACTs? | 1. Yes ………………………………....**🡪Go to Q224** 2. No……….……………………….....**🡪Go to Q225** | |
| 224 | | List the side effects for each ACT in the boxes given below?   | **Drug Name** | **Side Effects** | | --- | --- | |  |  | |  |  | |  |  | |  |  | |  |  | |  |  | |  |  | |  |  | |  |  | |  |  | |  |  | | | | |
| 225 | | Do you ever receive any information on ACTs? | | | 1. Yes ………………………………....**🡪Go to Q226** 2. No……….………………...……….....**🡪Go to Q227** |
| 226 | | Where do you get information on ACTs from? | | | 1. Books……………… …………………………………..... 2. Peer reviewed journals………………………….... 3. Drug formulary……………………………………..... 4. Malawi Government documents…………….... 5. Other (specify) ……………………………………….   …………………………………….…………………………….. |
| 227 | | Do you have the Ministry of Health guidelines on the treatment of malaria in Malawi? | | | 1. Yes ……………………………....**🡪Go to Q228** 2. No……….…………………….....**🡪Go to Q229** |
| 228 | | Where did you obtain the guidelines from? | | | ……………………………………………………………………  …………………………………………………………………… |
| 229 | | Do you receive any information on ACTs from pharmaceutical companies? | | | 1. Yes ……………………………....**🡪Go to Q230** 2. No……….…………………….....**🡪Go to Q231** |
| 230 | | Which pharmaceutical companies provide you with information on ACTs? | | | ………………………………………………………………… |
| 231 | | Have you ever received any training on management of malaria using ACTs since 2007? | | | 1. Yes ……………………………....**🡪Go to Q232** 2. No……….…………………….....**🡪Go to Q234** |
| 232 | | How long was the training?  **[Approximate number of days]** | | | ……………………………………………………………….... |
| 233 | | Who conducted the training?  [**The main organizer or organization responsible**] | | | ………………………………………………………………… |
| 234 | | Of all the sources of information on ACTs you have mentioned, which one do you find to be most useful? | | | …………………………………………………………………….  ……………………………..……………………………………. |

| **Module III: Pharmacovigilance** | | |
| --- | --- | --- |
| 301 | Have you ever had an incidence of an adverse event (AE)/serious adverse event (SAE) reported after prescribing ACTs? | 1. Yes ………………..………………………………………….... go to Q302  2. No ………………………….…………….…………………. go to Q304 |
| 302 | Which ACTs caused these AEs/SAEs? | 1. ……………………………………................................................ 2. …………………………………………….…………...………….... 3. …………………………………………………..………………….. 4. …………………………………………………….......................... 5. ………………………………………………................................. |
| 303 | For each of the ACTs that you have listed in Q302, how many times have you received reports of AEs/SAEs and over what time period? | | Name of ACTS | No. of AEs/SAEs | Time period (*months)* | | --- | --- | --- | |  |  |  | |  |  |  | |  |  |  | |  |  |  | |  |  |  | |
| 304 | Do you know who to contact when you get a report of an AE/SAE? | 1. Yes ………………..………………………………………….... go to Q305  2. No ………………………….………………………………. go to Q309 |
| 305 | Do you pass on the report to any national authority? | 1. Yes ………………..………………………………………….... go to Q306  2. No ………………………….…………………………………. go to Q307 |
| 306 | Which national authority do you pass the information to? | ……………………………………………………………………………………………… |
| 307 | Do you pass on the report to any importer? | 1. Yes ………………..………………………………………….... go to Q308  2. No ………………………….…………………………………. go to Q309 |
| 308 | Which importer do you pass the information to? | ……………………………………………………………………………………………… |
| 309 | Have you ever received any training on how to report AEs/SAEs? | 1. Yes ………………..………………………………………….... go to Q310  2. No ………………………….…………………………………. go to Q401 |
| 310 | Where did you receive the training?  [Tick all that apply] | 1. Medical council of Malawi ………………….…………………………. 2. Society of Medical Doctors, Malawi………………….…………….. 3. Pharmaceutical Industry ………………….…………………………... 4. Ministry of Health ………………….…………………………………….. 5. Research Institution………………….…………………………….……. 6. Other………………….………………………………………………………… |

| **Module III: Malaria Treatment Practices** | | |
| --- | --- | --- |
| 301 | Do you prescribe different ACTs to patients? | 1. Yes ………………..……………………………………………....  2. No ………………………….……………………………………. |
| 302 | How do you decide which treatment to prescribe to a patient?  **[Tick all that apply]** | 1. Based on age ………………................................................ 2. Based on the clinical picture……………...………….... 3. Based on availability of drugs……..………………….. 4. Based on diagnostic tests available.......................... 5. Based on the cost of the drugs .................................. 6. Other (specify)………………………………………………    ……………………………………………….. |
| 303 | Are there any guidelines available that you use when treating patients with malaria? | 1. Yes ………………..………………………………....  Indicate guidelines used below:  …………………………………………………………………………..…………  ………………………………………………………………………………..……  2. No …………………………………………. |

Are there any other comments you would like to give us.

__________________________________________________________________________________________________________________________________________________________________________________________________________________________________________________________________________________________________________________________________________________________________________________________________________________________________________________________________________________________________________________________________________________________________________________________________________________________________________________________________________________________________________________________________________________________________________________________________________________________________________________________________________________________________________________________________________________________________________________________________________________________________________________________________________________________________________________________________________________________________________

***END: Thank you for your cooperation***
